# Perspective: Simple State Communities to Study Microbial Interactions: Examples and Future Directions

**DOI:** 10.3389/fmicb.2022.801864

**Published:** 2022-01-27

**Authors:** Soumyadev Sarkar, Kaitlyn Ward, Abigail Kamke, Qinghong Ran, Brandi Feehan, Tanner Richie, Nicholas Reese, Sonny T. M. Lee

**Affiliations:** Division of Biology, Kansas State University, Manhattan, KS, United States

**Keywords:** 16S rRNA amplicon, microbes, interactions, simple state communities, metagenome

## Abstract

Microbial interactions in natural environments are intricately complex. High numbers and rich diversity of microorganisms, along with compositional heterogeneities complicate the cause. It is essential to simplify these complex communities to understand the microbial interactions. We proposed a concept of “*simple state community*,” which represents a subset of microbes and/or microbial functions of the original population that is necessary to build a stable community. By combining microbial culturing and high-throughput sequencing, we can better understand microbe-microbe and microbe-host interactions. To support our proposed model, we used carbon-based and nitrogen-based media to capture the simple state communities. We used 16S rRNA amplicon sequencing and assigned taxonomic identity to the bacterial populations before and after simple state communities. We showed that simple state communities were a subset of the original microbial communities at both phyla and genera level. We further used shotgun metagenomics to gain insights into the functional potential of the assembled simple state communities. Our proposed model supported the goal of simplifying the complex communities across diverse systems to provide opportunity to facilitate comprehension of both the structure and function of the subset communities. Further applications of the concept include the high-throughput screening of simple state communities using the BIOLOG^®^ system and continuous culturing (Chemostat). This concept has the potential to test diverse experimental hypotheses in simplified microbial communities, and further extend that knowledge to answer the overarching questions at a more holistic level.

## Introduction

From the soil under our feet to the corals in the ocean, and the millions of microbes in our gastrointestinal tract, microbes inhabit nearly every environment on Earth (Finlay and Esteban, 2001). The microbial composition and their functions change dynamically in response to their local environment as well as specific parameters such as water chemistry, light, temperature, salinity, chemical and biological factors (Koranda et al., 2013; Deaver et al., 2018; Rath et al., 2019; Zhou et al., 2020). As the number of microbial groups within a particular environment often exceeds several thousand distinct taxonomic clades (Microbiology by numbers, 2011), there is a considerable push in the scientific community in using simplified microbial communities to test hypotheses (Garcia, 2016; Niu et al., 2017; Nai and Meyer, 2018; Yu et al., 2019; Pascual-García et al., 2020; Kapoore et al., 2021; Xu and Yu, 2021). Currently, there are two popular approaches for capturing the subset communities – “top-down” and “bottom-up.” More attempts till now have followed the synthetic communities generation through “bottom-up” approaches where various isolates are inoculated together to reconstruct the whole community (Venturelli et al., 2018; Carlström et al., 2019), but this approach has to overcome challenges of determining which isolates will co-exist and function together. In this study, we followed the less explored “top-down” approach where we use the concept of serial dilution to capture the simplified communities. Although both approaches have certain advantages and disadvantages, there have been strong arguments that “top-down” approaches are better able to mimic the way diversity affects the natural environment’s community function (Yu et al., 2019; Pascual-García et al., 2020).

As microbes inhabit various environments, it is essential to understand how microbial communities maintain their stability. Therefore, it is crucial to create approaches for minimizing the complexity of natural microbial communities to allow for a deeper understanding of the microbial interactions. Present non-cultivation-based methods study whole-scale microbial communities that are built upon coarse sampling methods and do not facilitate functional understanding of member organisms. On the other hand, individual collections of isolates in the laboratory do not provide insights into the role of microbial communities in the environment. Therefore, we proposed the creation of a “*simple state community*” of the microbiome in the laboratory from a variety of environments, to enable us to understand the building blocks of stability in these systems and facilitate the further investigation of the microbe-microbe and host-microbe interactions. We defined “*Simple State Community”* as a subset of microbes and/or microbial functions of the original population that is required to build a stable community (de Vos, 2013). The term “state” here is referred to the stable community that is captured targeting specific collective microbial function, such as nitrogen fixation or carbohydrate utilization. By “*simplifying*” the native microbial community through enrichment in the laboratory, we can remove the noise from the myriad of co-existing metabolic and functional pathways that are present in the environment. This will enable us to focus on a specific process of interest which can answer questions of interest in microbial ecology using modern omics technology.

This *“simple state”* approach is frequently used to examine a subset of native communities such as sulfate reducers, nitrate denitrifiers, or micro-organisms that are capable of degrading specific compounds of interest (Niu et al., 2017). Although studies using “*simple state communities”* are not uncommon, this approach together with high-throughput sequencing could provide greater opportunities to gain insights into how microbial community members interact with each other, their environments, and their host.

We proposed a concept of a reductionist approach – the “*simple state community*” (SSC). This would include targeted screening, cultivation, and monitoring platforms. The concept drives in the direction to capture the sub-communities (SSC) so that defined systems can be generated and further analyzed by transcriptome, proteome, metabolome, or other approaches. We used the word SSC to refer to the sub-community because it is a simplified subset based on targeted substrate utilization of the original complex community (pre-SSC). We proposed substrate utilization as a primary screen and then monitor the expression of the sub-communities. In this way, responses of multiple organisms and clades, distinguished through the initial screen, can be recorded with changing dynamics and complexity of the system, resulting in more clarity on the functional aspect of the communities toward the host.

The approach of SSC will not only allow the relatively easy study of complex communities in the laboratory but also would help to monitor if any defined biotic and/or abiotic disturbances are taking place on the population over time. Integration of all these data together would be perfect to understand the complexities.

## A Simple State Approach to Study Complex Microbiome Communities

**FIGURE 1:**
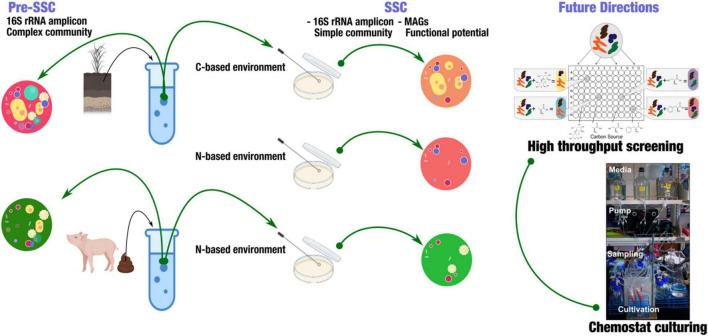
Experimental strategy to obtain simple state communities (SSC) from soil and fecal samples. This strategy highlighted obtaining a simple, targeted community that enabled the further investigation of functional potential in metagenome-assembled genomes (MAGs). Further applications include high-throughput screening of SSC using BIOLOG^®^ system and continuous culturing (Chemostat).

The basic goal for our SSC model is that to provide a framework to dissect complex microbial community members in a high-throughput manner (Figure 1). Here, we produced a summary of sampling strategies using 16S rRNA amplicon to analyze samples from soil and swine gut. The goal of this summary is to provide insights into the use of SSC to identify a subset of microbial communities, and how this could be applied in the future to understand the novel microbial interactions. To demonstrate the feasibility of this model, we used soil from Western Kansas and swine fecal samples (gut). We used both carbon and nitrogen-based media to cultivate SSCs from soils, while fecal SSCs were obtained from a nitrogen-limited based environment. We used 16S rRNA amplicon to identify the pre-SSC and SSC communities.

### Culture Conditions, Cultivation, DNA Extraction, 16S Amplicon Sequencing, and Bioinformatics Analysis

We wanted to demonstrate that our proposed model could be applied to a wide range of samples such as soil and fecal, as well as in different conditions such as low moisture environments. R2A agar plates were used as the carbon-based medium that contained ample sources of nutrients for bacteria to thrive on (de Raad et al., 2021). On the other hand, we prepared nitrogen-limiting medium which contained (per liter): K_2_S0_4_ (1 g), KH_2_PO_4_ (4.7 g), K_2_HPO_4_ *3H_2_0 (17.7 g), MgSO_4_ – 7H_2_0 (0.1 g), NaCl (2.5 g), glucose (4 g) as the carbon source, and NH_4_Cl (1 mM concentration) as the nitrogen source (Gutnick et al., 1969). Polyethylene Glycol (36% w/v) was appended to the carbon-based medium to induce a low-moisture condition and generate an environmental pressure (Marulanda et al., 2009). The soil (1 g of soil was added to 9 ml of ultra-pure water) and fecal (0.1 g was added to 0.9 ml of ultra-pure water) samples were serially diluted (10^–1^ – 10^–6^) and spread on the Petri plates. We incubated the plates at 37°C for 48 h to enable optimal growth of distinct colonies from both the soil and fecal samples. The samples were cultured in Petri-plates for this study as the nutrient cross-feeding interactions are well observed when microbes are isolated on conventional agar plates (Abreu and Taga, 2016), and also that the agar plates have been reported to capture as much as 50% bacterial OTUs from stool samples (Goodman et al., 2011). Plates with countable and distinct colonies were all scrapped into phosphate-buffered saline (PBS) [pH 7] for microbial DNA extraction for 16S rRNA amplicon. We were interested in capturing the subset of the complex communities that were growing together along with the functional potential, thus we did not pick colonies to sequence the full genome but instead, we scrapped all colonies from the plates to sequence 16S rRNA amplicons. All the experiments were performed in triplicates.

We extracted the microbial DNA from the SSC colonies and soil samples using the E.Z.N.A. Soil DNA Kit (Omega Bio-Tek, Inc., Norcross, GA, United States) following the manufacturer’s protocol. We used the E.Z.N.A Stool DNA Kit (Omega Bio-Tek, Inc., Norcross, GA, United States) to extract microbial DNA directly from stool samples following the manufacturer’s protocol. Extracted DNA was sequenced on an Illumina MiSeq platform to profile 16S rRNA V4 amplicons using 515F and 806R [515F-GTGCCAGCMGCCGCGGTAA and 806R-GGACTACHVGGGTWTCTAAT] primers with appropriate barcodes (Illumina, San Diego, CA, United States). We used Qiime 2 v2019.10 (Bolyen et al., 2019) to process the sequences and profiled the soil and fecal community structure. Briefly, we trimmed the primers and adaptors, and used DADA2 to extract precise amplicon sequence variants (ASVs) from the sequences (Callahan et al., 2016). We used the SILVA database for classifying ASVs taxonomically (Quast et al., 2013). Shannon, Faith PD and observed OTUs indices were used to analyze the species diversity. Community dissimilarity patterns were evaluated by UniFrac and Bray Curtis. We used Non-metric multidimensional scaling (NMDS) to visualize distance matrices. For shotgun metagenomes, the samples were sequenced on the Illumina NovaSeq 6000 (Illumina, San Diego, CA, United States), using the S1 flow cell with 150 paired-end sequencing strategy. Detailed bioinformatic protocol for processing of the metagenomes is attached as a Supplementary Methods 1. Briefly, we assembled the quality-filtered short reads into longer contiguous sequences (contigs) and identified open reading frames (ORFs). Following that, we recruited metagenomic short reads to the contigs, and binned metagenome-assembled genomes (MAGs). We manually curated these MAGs to ensure they accurately represented microbial populations. We then assigned taxonomy to the MAGs using sets of bacterial and archaeal single-copy core genes. We assigned functions to the ORFs using the NCBI’s Clusters of Orthologous Groups (COGs).

#### Identification of Simple State Community Members

We observed that following incubation and media screening, the SSC structure revealed a simpler subset of the pre-SSC microbial community. We observed that the SSC members were representative of the parent community both at the phyla level as well as the genera level. These compositions were also reproducible in the replicated samples (Supplementary Table 1). We believe representation and reproducibility are crucial in order for the top-down simplified microbial communities’ approach to be useful and be implemented in other studies as well.

**FIGURE 2:**
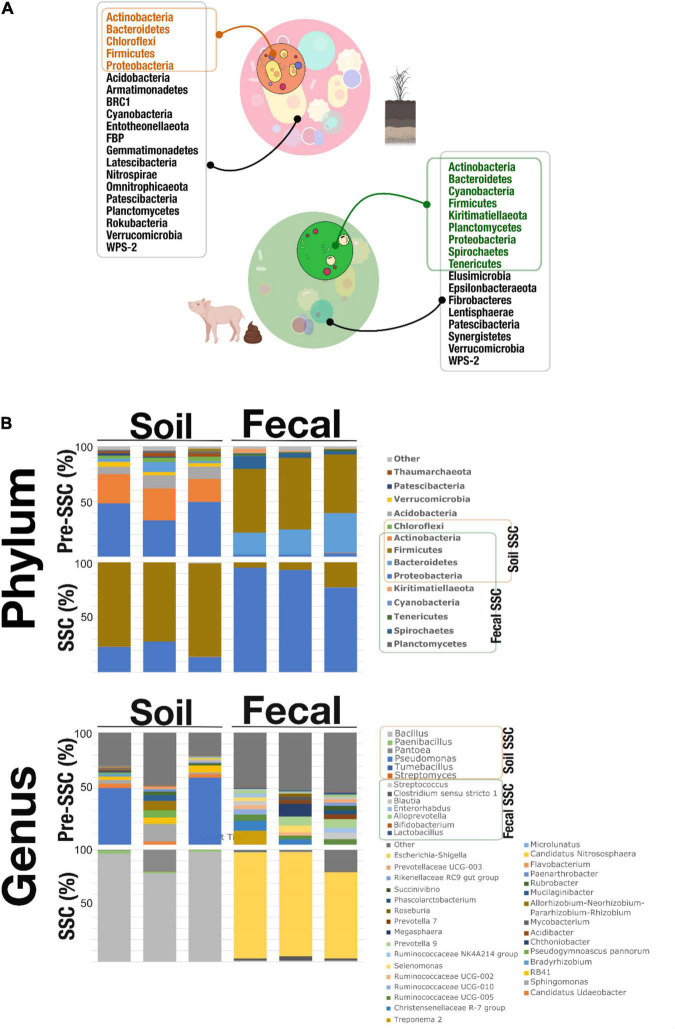
**(A)** SSC members (phyla) that were recovered through the SSC strategy. The soil SSC structure was able to capture Actinobacteria, Bacteroidetes, Chloroflexi, Firmicutes, and Proteobacteria which overlapped with the pre-SSC. Fecal SSC identified Actinobacteria, Bacteroidetes, Cyanobacteria, Firmicutes, Kiritimatiellaeota, Planctomycetes, Proteobacteria, Spirochetes, and Tenericutes that were also present in the fecal pre-SSC structure. This proved that the SSC strategy was able to simplify complex communities from different systems (soil and fecal) at the level of phyla. **(B)** Stacked bar plots showing the relative abundance of the microbial community, at the phylum and genus level, in pre-SSC and SSC soil and fecal samples.

Figure 2 showed the microbial composition on the phylum and genus level in the pre-SSC and SSC soil and fecal samples. We noticed that the SSC soil samples represented 31.81% of the original community. Actinobacteria, Bacteroidetes, Chloroflexi, Firmicutes, and Proteobacteria were found in both the pre-SSC and SSC soil samples (Figures 2A,B and Supplementary Table 1). In the Pre-SSC soil samples, *Pseudomonas* (relative abundance; mean ± standard deviation: 0.25 ± 0.15) and *Micrococcaceae* (0.14 ± 0.02) were the most dominant bacterial genera. For the soil SSC structure, there was a dominance of *Bacillus* (0.76 ± 0.05) followed by *Enterobacteriaceae* (0.16 ± 0.05) and *Paenibacillus* (0.01 ± 0.003). The common genera between pre-SSC and SSC structures included *Streptomyces*, *Dehalococcoidia*, *Chloroflexi KD4-96*, *Tumebacillus*, *Bacillus*, *Pantoea*, *Enterobacteriaceae*, *Pseudomonas*, and *Paenibacillus* (Figure 2B and Supplementary Table 1).

Similarly, fecal SSC showed a recovery of bacterial populations of interest that was a subset (55.55%) of the complex fecal community (Figure 2A). At the phylum level, Actinobacteria, Bacteroidetes, Cyanobacteria, Firmicutes, Kiritimatiellaeota, Planctomycetes, Proteobacteria, Spirochetes, and Tenericutes were present in both the pre-SSC and SSC structure (Figures 2A,B and Supplementary Table 1). Some of the common genera between pre-SSC and SSC fecal structures included *Bifidobacterium*, *Enterorhabdus, Alloprevotella, Prevotella, Lactobacillus, Streptococcus, Clostridium sensu stricto 1, Blautia*, and *Coprococcus*, among others (Figure 2B and Supplementary Table 1). The fecal SSC samples showed the highest relative abundance of *Escherichia-Shigella* (0.88 ± 0.07), followed by *Clostridium sensu stricto 1* (0.02 ± 0.01) and *Streptococcus* (0.01 ± 0.002) (Figure 2B).

In the preliminary study for our proposed model, we demonstrated a significant reduction in the complexity of the SSC as compared to the original community. We used the Kruskal-Wallis statistical analyses and showed statistical differences in the bacterial α-diversity between the SSCs and the original communities (S_Obs_, Shannon index: *H* = 9.46, *p* = 0.02; Faith PD index: *H* = 9.46, *p* = 0.02; observed OTUs: *H* = 9.7, *p* = 0.02; Supplementary Figure 1). Similarly, there was distinct clustering of the original communities and SSC samples (soil and fecal) at both phyla and genera levels (Supplementary Figure 2). We used PERMANOVA statistical analyses and showed that there were significant differences between soil Pre-SSC and SSC community composition {[Phylum: PERMANOVA, Pseudo-F 145.96, p (MC): 0.001]; Genus: PERMANOVA, Pseudo-F 26.585, p (MC): 0.002} as well as fecal Pre-SSC and SSC microbial community {[Phylum: PERMANOVA, Pseudo-F 90.663, p (MC): 0.001];.

#### Functional Potential of Simple State Community Members

One of the advantages of obtaining SSCs from a complex microbial community such as the soil environment is the high possibility of assembling metagenome-assembled genomes/draft bacterial genomes from the cultivation, allowing for further investigation of the functional potential of specific bacterial populations. SSC provided the opportunity to further use various annotation tools and databases such as DRAM (Distilled and Refined Annotation of Metabolism) (Shaffer et al., 2020) and The database of Clusters of Orthologous Groups (COGs) of proteins to gain insights into the functional profiles of the assembled genomes (Tatusov et al., 2000).

Beyond taxonomic identity that was provided by 16S amplicon sequences, we assigned gene functional potentials to the MAGs using numerous databases. We recovered 27 highly resolved MAGs from carbon-based SSC treatment from the soil samples. We also recovered 37 MAGs from carbon-based treatment appended with Polyethylene Glycol. On the other hand, we recovered only two and eight MAGs from the more nutrient-limited, nitrogen-based SSC treatment for both the fecal and soil samples, respectively (Supplementary Table 2). We used DRAM functional annotation tool, and reported microbial carbon and nitrogen utilization functional potential as well as genes responsible for energy transduction and transport systems. We further classified genes responsible for rRNA and tRNA, Electron Transport Chain (ETC) complexes, Carbohydrate-Active enzymes, nitrogen metabolism, Short Chain Fatty Acids (SCFA), and alcohol conversions (Supplementary Figure 3 and Supplementary Table 2).

## Conclusion and Future Perspective

Comparing the Pre-SSC and SSC microbial populations, we showed that the SSC communities were a subset of the Pre-SSC communities in the level of phyla and genera. Furthermore, the possibility of elucidating the functional potential of the simplified community indicated that information about nutrient utilization can be deduced. This supported our goal in simplifying the complex communities from different systems (soil and fecal) that would help toward easier understanding of both the structure and the function of the subset communities. In validating this approach, we utilized Petri plates as the environment in which different media could be designed and allowed only certain microbial communities to grow. The proposed framework simplified microbial community complexity as well as laid a foundation to apply the concept by conjugating with high-throughput plates and chemostats.

This concept is to be implemented using high-throughput plates to simultaneously study a high number of samples along with their replicates. BIOLOG^®^ microplates are well-suited for this cause; coupling these microplates with different types of nutrient source utilization is useful to identify subsets of the initial complex community (Figure 1).

Chemostats to be used as a tool to establish SSC communities (Figure 1). Chemostats use the principle of the constant influx of nutrients and an equivalent efflux of biomass. As the flow rate remains constant, so does the cell growth rate. Likewise, any changes in the response of the organism can be tracked under any disturbances. In our SSC model, with appropriate adaptation, we aimed to link this conjugate of highly parallel continuous cultures and substrate utilization panels in the BIOLOG^®^ with Next-Generation Sequencing (NGS) technologies (Figure 1). The goal is to capture the subsets of the primary community according to the screening of substrate utilization. We then could characterize the microbe-microbe interactions and changes that are due to abiotic and/or biotic disturbances in the continuous cultures (Miller et al., 2013). This concept aims at targeting specific communities based on certain microbial functions and substrate utilization, enabling us to investigate only the taxa that we were interested in. The SSC model simplified community complexity, providing the opportunity to better comprehend microbial community stability and functioning.

As a final point, one should keep in mind that the targeted media approach in our SSC model would result in capturing microbial members that are highly competitive for space and nutrients. Careful planning of future studies, taking into consideration the biases and pitfalls of culture-based assays is necessary.

## Data Availability Statement

The original contributions presented in the study are included in the article/Supplementary Material, further inquiries can be directed to the corresponding author.

## Author Contributions

SS conceptualized, performed the experiments, analyzed the data, and wrote the manuscript. KW and AK performed the experiments. QR analyzed the data. BF, TR, and NR analyzed the data and reviewed the writing for the manuscript. SL conceptualized, supervised, analyzed the data, was responsible for resource acquisitions, and wrote the manuscript. All authors contributed to the article and approved the submitted version.

## Conflict of Interest

The authors declare that the research was conducted in the absence of any commercial or financial relationships that could be construed as a potential conflict of interest.

## Publisher’s Note

All claims expressed in this article are solely those of the authors and do not necessarily represent those of their affiliated organizations, or those of the publisher, the editors and the reviewers. Any product that may be evaluated in this article, or claim that may be made by its manufacturer, is not guaranteed or endorsed by the publisher.

## Supplementary Material

Supplementary Figure 1 Bacterial α-diversity (Shannon, Faith’s PD and Observed OTUs indices) in Pre-SSC and SSC communities in soil and fecal samples.

Supplementary Figure 2 NMDS visualization of Pre-SSC and SSC microbial communities in soil and fecal samples.

Supplementary Figure 3 Analysis of MAGs by DRAM generates a profile that can be utilized to compare functions across complexes, metabolic and enzymatic pathways. ETC complex I: ubiquinone oxidoreductase, ETC complex II: succinate dehydrogenase, ETC complex III: cytochrome c reductase, ETC complex IV: cytochrome c oxidase, ETC complex V: ATP synthase. Environmental pressure for the carbon-based SSC here refers to the use of Polyethylene Glycol (36% w/v) to the carbon-based medium to induce a low-moisture condition.

Supplementary Table 1 Taxonomic assignment of ASVs and counts (Kingdom, Phylum, Class, Order, Genus, Species) in pre-SSC and SSC from soil and fecal samples based on 16S rRNA V4 amplicon.

Supplementary Table 2 Metagenome-assembled genomes (MAGs) statistics – total length, number of contigs, N50, GC contents, completion and redundancy percentage, and summary of functional potential.

Supplementary Methods 1 Metagenomic analyses methods.
