# Supplementary figures and images for: Perspective: Simple State Communities to Study Microbial Interactions: Examples and Future Directions
Source: Front Microbiol. 2022 Jan 27;13:801864. doi: 10.3389/fmicb.2022.801864 (PMC8828649; doi:10.3389/fmicb.2022.801864)

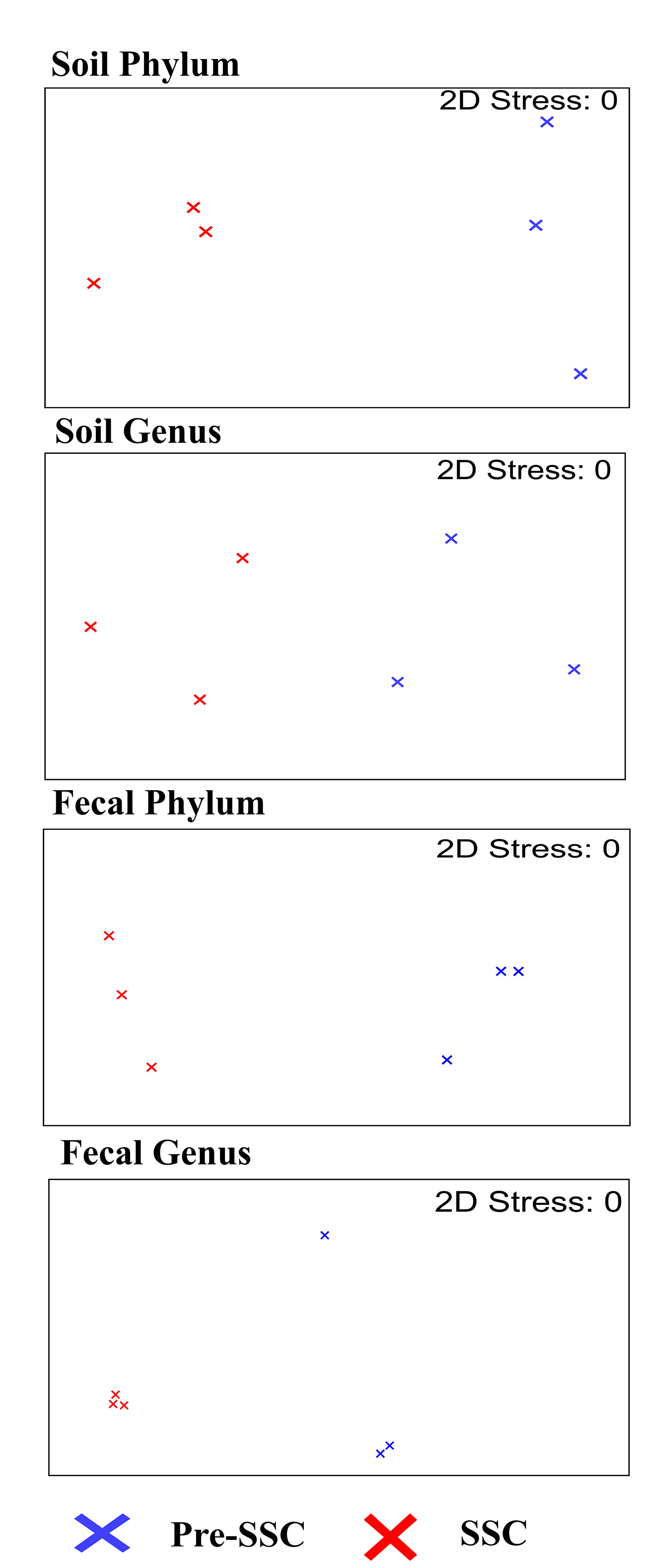

Supplement: Supplementary Figure 1 — Bacterial α-diversity (Shannon, Faith’s PD and Observed OTUs indices) in Pre-SSC and SSC communities in soil and fecal samples. [file Image_1.JPEG]

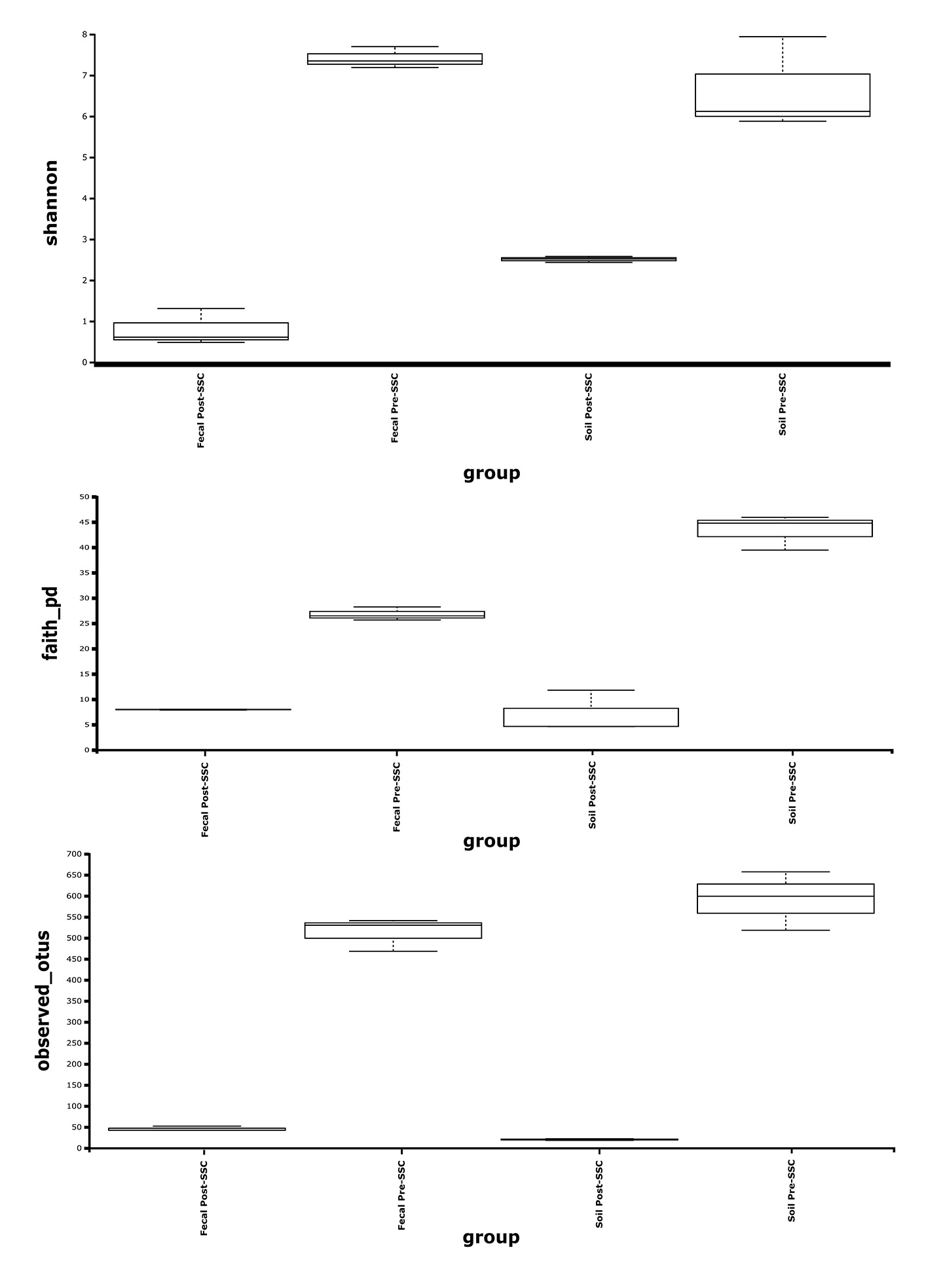

Supplement: Supplementary Figure 2 — NMDS visualization of Pre-SSC and SSC microbial communities in soil and fecal samples. [file Image_2.JPEG]

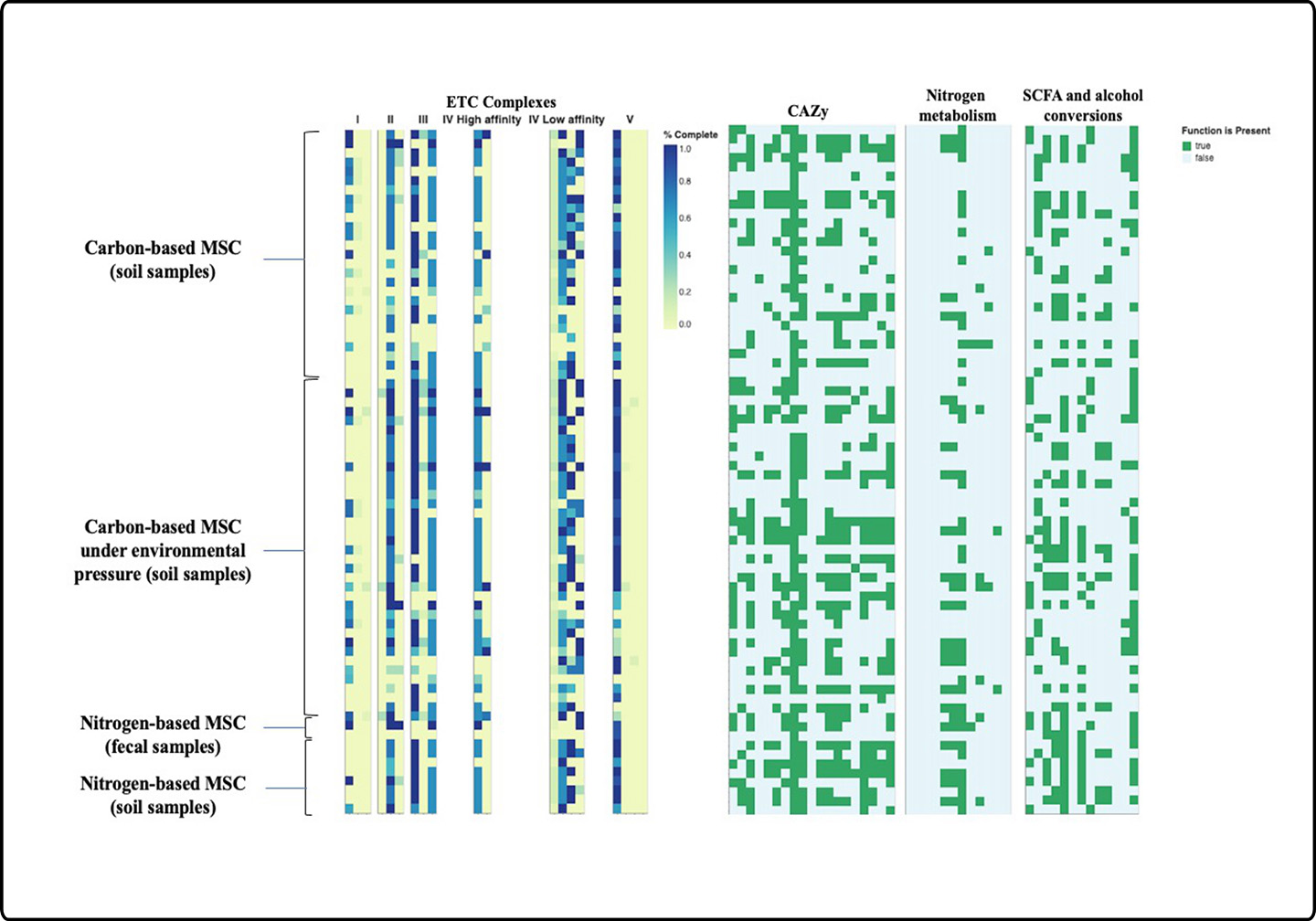

Supplement: Supplementary Figure 3 — Analysis of MAGs by DRAM generates a profile that can be utilized to compare functions across complexes, metabolic and enzymatic pathways. ETC complex I: ubiquinone oxidoreductase, ETC complex II: succinate dehydrogenase, ETC complex III: cytochrome c reductase, ETC complex IV: cytochrome c oxidase, ETC complex V: ATP synthase. Environmental pressure for the carbon-based SSC here refers to the use of Polyethylene Glycol (36% w/v) to the carbon-based medium to induce a low-moisture condition. [file Image_3.JPEG]
