# Supplementary material for: Perspective: Simple State Communities to Study Microbial Interactions: Examples and Future Directions
Source: Front Microbiol. 2022 Jan 27;13:801864. doi: 10.3389/fmicb.2022.801864 (PMC8828649; doi:10.3389/fmicb.2022.801864)
Supplement: Supplementary Methods 1 — Metagenomic analyses methods. [file Table_3.docx]

**Supplementary Methods 1**

**Supplementary metagenomic analyses methods**

The samples were sequenced on the Illumina NovaSeq S1 flow cell with 150 paired-end sequencing strategy. Whenever applicable, we automated our ‘omics analyses using the bioinformatics workflows implemented by the program `anvi-run-workflow` (Shaiber et al. 2020) in anvi’o (Eren et al. 2015). Anvi’o workflows implement various bioinformatics tasks including short-read quality filtering, assembly, gene calling, functional annotation, hidden Markov model search, metagenomic read-recruitment, and metagenomic binning. Workflows use Snakemake (Köster and Rahmann 2018) and the following sections detail these steps:

**Assembly of metagenomic short reads.** To minimize the impact of random sequencing errors in our downstream analyses, we used the program `iu-filter-quality-minoche` to process short metagenomic reads, which is implemented in illumina-utils v2.11 (Eren et al. 2013) and removed low-quality reads according to the criteria outlined by Minoche et al. [(Minoche, Dohm, and Himmelbauer 2011)](https://paperpile.com/c/Qzu8VF/pMhCY). Megahit (D. Li et al. 2015) assembled quality-filtered short reads into longer contiguous sequences (contigs).

**Processing of contigs.** We used the following strategies to process sequences we obtained from our assemblies. We used (1) `anvi-gen-contigs-database` on contigs to compute k-mer frequencies and identify open reading frames (ORFs) using Prodigal v2.6.3 (Hyatt et al. 2010), (2) `anvi-run-hmms` to identify sets of bacterial (Campbell et al. 2013) and archaeal (Rinke et al. 2013) single-copy core genes using HMMER v3.2.1 (Eddy 2011), (3) `anvi-run-ncbi-cogs` to annotate ORFs with functions from the NCBI’s Clusters of Orthologous Groups (COGs) (Tatusov et al. 2003).

**Metagenomic read recruitment, reconstructing genomes from metagenomes, determination of genome taxonomy.** We recruited metagenomic short reads to contigs using Bowtie2 v2.3.5 (Langmead and Salzberg 2012) and converted resulting SAM files to BAM files using samtools v1.9 (H. Li et al. 2009). We profiled the resulting BAM files using the program `anvi-profile`, and used `anvi-merge` combined all profiles into an anvi’o merged profile for downstream visualization, binning, and statistical analyses. We used `anvi-cluster-contigs` to group contigs into bins using CONCOCT v1.1.0 (Alneberg et al. 2013), and `anvi-refine` to manually curate initial bins with conflation error based on tetranucleotide frequency and differential coverage signal across all samples. We identified bins that were more than 70% complete and less than 10% redundant, and stored them in a new collection as metagenome-assembled genomes (MAG), discarding lower quality bins from downstream analyses. To assign taxonomy, we used `anvi-get-sequences-for-hmm-hits` to recover DNA sequences for bacterial single-copy core genes that encode ribosomal proteins, and searched them in the NCBI’s nucleotide collection (nt) database using BLAST (Altschul et al. 1990).

**DRAM (Distilled and Refined Annotation of Metabolism).** We used DRAM tool to gain insights into the functional profiles of the MAGs. We recovered 27 highly resolved MAGs from carbon-based MSC treatment from the soil samples. We also recovered 37 MAGs from carbon-based treatment appended with Polyethylene Glycol. On the other hand, we recovered only two and eight MAGs from the more nutrient-limited, nitrogen-based MSC treatment for both the fecal and soil samples respectively (Supplementary Table S2). We reported microbial carbon and nitrogen utilization functional potential as well as genes responsible for energy transduction and transport systems. We further classified genes responsible for rRNA and tRNA, Electron Transport Chain (ETC) complexes, Carbohydrate-Active enzymes, nitrogen metabolism, Short Chain Fatty Acids (SCFA), and alcohol conversions (Fig. 2B).

**References**

Alneberg, Johannes, Brynjar Smari Bjarnason, Ino de Bruijn, Melanie Schirmer, Joshua Quick, Umer Z. Ijaz, Nicholas J. Loman, Anders F. Andersson, and Christopher Quince. 2013. “CONCOCT: Clustering CONtigs on COverage and ComposiTion.” *ArXiv [q-Bio.GN]*. arXiv. http://arxiv.org/abs/1312.4038.

Altschul, S. F., W. Gish, W. Miller, E. W. Myers, and D. J. Lipman. 1990. “Basic Local Alignment Search Tool.” *Journal of Molecular Biology* 215 (3): 403–10.

Campbell, James H., Patrick O’Donoghue, Alisha G. Campbell, Patrick Schwientek, Alexander Sczyrba, Tanja Woyke, Dieter Söll, and Mircea Podar. 2013. “UGA Is an Additional Glycine Codon in Uncultured SR1 Bacteria from the Human Microbiota.” *Proceedings of the National Academy of Sciences* 110 (14): 5540–45.

Eddy, Sean R. 2011. “Accelerated Profile HMM Searches.” *PLoS Computational Biology* 7 (10): e1002195.

Eren, A. Murat, Özcan C. Esen, Christopher Quince, Joseph H. Vineis, Mitchell L. Sogin, and Tom O. Delmont. 2015. “Anvi’o: An Advanced Analysis and Visualization Platform for ‘omics Data.” *PeerJ*. https://doi.org/10.7287/peerj.preprints.1275.

Eren, A. Murat, Joseph H. Vineis, Hilary G. Morrison, and Mitchell L. Sogin. 2013. “A Filtering Method to Generate High Quality Short Reads Using Illumina Paired-End Technology.” *PloS One* 8 (6): e66643.

Hyatt, Doug, Gwo-Liang Chen, Philip F. LoCascio, Miriam L. Land, Frank W. Larimer, and Loren J. Hauser. 2010. “Prodigal: Prokaryotic Gene Recognition and Translation Initiation Site Identification.” *BMC Bioinformatics* 11 (1): 1–11.

Köster, Johannes, and Sven Rahmann. 2018. “Snakemake-a Scalable Bioinformatics Workflow Engine.” *Bioinformatics (Oxford, England)* 34 (20): 3600.

Langmead, Ben, and Steven L. Salzberg. 2012. “Fast Gapped-Read Alignment with Bowtie 2.” *Nature Methods* 9 (4): 357–59.

Li, Dinghua, Chi-Man Liu, Ruibang Luo, Kunihiko Sadakane, and Tak-Wah Lam. 2015. “MEGAHIT: An Ultra-Fast Single-Node Solution for Large and Complex Metagenomics Assembly via Succinct de Bruijn Graph.” *Bioinformatics*  31 (10): 1674–76.

Li, Heng, Bob Handsaker, Alec Wysoker, Tim Fennell, Jue Ruan, Nils Homer, Gabor Marth, Goncalo Abecasis, Richard Durbin, and 1000 Genome Project Data Processing Subgroup. 2009. “The Sequence Alignment/Map Format and SAMtools.” *Bioinformatics*  25 (16): 2078–79.

Rinke, Christian, Patrick Schwientek, Alexander Sczyrba, Natalia N. Ivanova, Iain J. Anderson, Jan-Fang Cheng, Aaron Darling, et al. 2013. “Insights into the Phylogeny and Coding Potential of Microbial Dark Matter.” *Nature* 499 (7459): 431–37.

Shaiber, Alon, Amy D. Willis, Tom O. Delmont, Simon Roux, Lin-Xing Chen, Abigail C. Schmid, Mahmoud Yousef, et al. 2020. “Functional and Genetic Markers of Niche Partitioning among Enigmatic Members of the Human Oral Microbiome.” *Genome Biology* 21 (1): 292.

Tatusov, Roman L., Natalie D. Fedorova, John D. Jackson, Aviva R. Jacobs, Boris Kiryutin, Eugene V. Koonin, Dmitri M. Krylov, et al. 2003. “The COG Database: An Updated Version Includes Eukaryotes.” *BMC Bioinformatics* 4 (September): 41.
